# Loss of minichromosome maintenance 4 leads to early renal dysplasia and cystogenesis outcomes

**DOI:** 10.1016/j.gendis.2025.101968

**Published:** 2025-12-08

**Authors:** Jing Huang, Hui-Ling Xiang, Qian Yuan, Chun Zhang, Xian-Fang Meng

**Affiliations:** a Department of Geriatrics, Tongji Hospital, Tongji Medical College, Huazhong University of Science and Technology, Wuhan, Hubei 430022, China; b Key Laboratory of Vascular Aging, Ministry of Education, Tongji Hospital, Tongji Medical College, Huazhong University of Science and Technology, Wuhan, Hubei 430022, China; c Department of Nephrology, Union Hospital, Tongji Medical College, Huazhong University of Science and Technology, Wuhan, Hubei 430022, China; d Department of Neurobiology, Institute of Brain Research, School of Basic Medical Sciences, Tongji Medical College, Huazhong University of Science and Technology, Wuhan, Hubei 430030, China

Renal dysplasia is defined by defective ureteric branching morphogenesis and nephrogenesis, which is the leading cause of renal failure in children. Currently, there is no effective therapeutic strategy.^1^ It is generally categorized as the complete absence of the kidneys, small kidneys (unilateral or bilateral) with or without normal renal architecture, and massive multicystic kidneys at the gross level.^2^ However, the detailed phenotypic features and regulatory mechanisms involved in the genesis of renal dysplasia remain poorly understood. Thus far, approximately 70 genes, including transcription factors (Six1 and Pax2) and growth factors (GDF11 and FGF), have been reported to play important roles in human renal dysplasia.^3^ In this study, we revealed the new genetic role of minichromosome maintenance complex component 4 (MCM4), highlighting the importance of MCM4 in maintaining tubular cell structure and kidney function. Deletion of MCM4 in kidney tubules resulted in fewer nephrons and a severely dysmorphic and dysplastic tubule system. MCM4 may be extended to identified as a new genetic target in renal development and provide valuable insights into potential therapeutic strategies and precision medicine.

MCM4 belongs to the MCM family and is characterized as a catalytic core of eukaryotic replicative helicases that are involved in DNA replication and genome stability.^4^ Previously, MCM4 was identified as an unknown oncogenic gene owing to the presence of rare genetic mutations. It has been reported that the loss of MCM4 appears to be associated with autosomal recessive syndrome of growth retardation, adrenal insufficiency, lymphoma, viral infections, and selective natural killer cell deficiency.^5^ However, the functional role of MCM4 in kidney disease remains largely unknown. To investigate the role of MCM4 in kidney development, tubule-specific MCM4 knockout mice were generated using a Cre-LoxP recombination system (Fig. S1). Although all the mice were born at a normal Mendelian ratio, 35 of the 40 Cre^+^/MCM4^fl/fl^ mice died within 40 days of birth, and most died within 2 weeks of birth; however, no deaths were observed among the Cre^−^/MCM4^fl/fl^ mice after birth (Fig. 1A). Compared to Cre^−^/MCM4^fl/fl^ mice, the surviving Cre^+^/MCM4^fl/fl^ mice seemed thinner (Fig. 1B) with lower body weight (Fig. 1C); however, the levels of serum creatinine and blood urine nitrogen were remarkedly increased (Fig. 1D). But the levels of alanine aminotransferase and aspartate aminotransferase were approximately the same in both groups (Fig. S2A). Morphologically, genetic deletion of MCM4 in tubules resulted in severe bilateral renal dysplasia, including the reduced size of the bilateral kidney, unilateral renal agenesis, and cystogenesis (Fig. 1E). But there were no notable lesions in the heart, liver, lung, or spleen of the mice in the two groups (Fig. S2B). These results revealed that MCM4 deficiency leads to kidney dysfunction and dysplasia, which may account for premature death in Cre^+^/MCM4^fl/fl^ mice.

**Figure 1:**
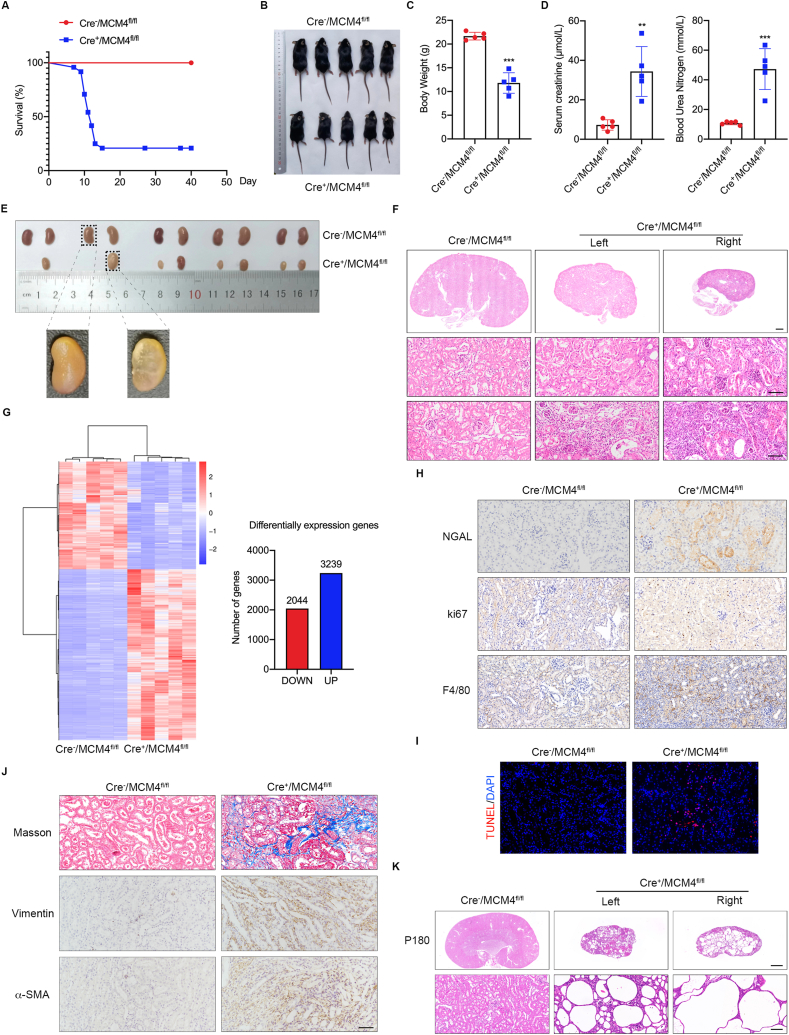
MCM4 was identified as a possible regulator of renal dysplasia and cystogenesis. **(A)** Survivorship curve of the mice in two groups at 40 days. **(B)** Gross appearance of the body of the mice in two groups at 40 days. **(C)** Graphical representation of the body weight in two groups at 40 days. **(D)** Graphical representation of the levels of serum creatinine and blood urea nitrogen in two groups at 40 days. **(E)** Gross appearance of the kidneys of the mice in two groups at 40 days. **(F)** Hematoxylin and Eosin (HE) staining of the kidneys of the mice in two groups at 40 days. Upper scale bar: 500 μm, lower scale bar: 50 μm. **(G)** Heatmap of differentially expressed genes in MCM4 knockout mice determined using RNA-seq analysis. **(H)** Representative immunostaining and summarized data showing the renal expression of NGAL, Ki67, and F4/80 in two groups of mice at 40 days, as indicated. Scale bar: 50 μm. **(I)** Representative images depicting TUNEL-positive cells in two groups of mice at 40 days. Scale bar: 50 μm. **(J)** Representative micrograph showing Masson's trichrome staining, and immunostaining of α-SMA and vimentin in two groups of mice at 40 days, as indicated. Scale bar: 50 μm. **(K)** HE staining showing cystic kidneys in Cre^+^/MCM4^fl/fl^ mice at postnatal month 6. Upper scale bar: 500 μm, lower scale bar: 50 μm. *n* = 5 for each group. All the data are presented as mean ± SEM. ∗∗*P* < 0.01, ∗∗∗*P* < 0.001 *vs.* Cre^−^/MCM4^fl/fl^.

Histologically, dysplastic kidneys are characterized by a paucity of nephrons, decreased branching morphogenesis, abnormal differentiation of mesenchymal and epithelial-derived tissue elements, and abnormal cortico-medullary patterning.^3^ Immunofluorescence (IF) staining of LTL, a proximal tubular marker, revealed a reduced number of tubules and destroyed tubular structure in the kidney cortex of Cre^+^/MCM4^fl/fl^ mice (Fig. S3A). IF staining of E-cadherin, an epithelial cell marker, further clarified the loss of tubular epithelial cell integrity and the disruption of the tubular basement membrane (TBM) in Cre^+^/MCM4^fl/fl^ mice (Fig. S3B). Compared to that in the wild-type mice, the area of the kidney in Cre^+^/MCM4^fl/fl^ mice was markedly reduced in cross-sections of the kidney (Fig. 1F). Hematoxylin and eosin staining also showed diffuse tubular swelling and disruption, destroyed tubular structure, denudation of the tubular basement membrane, and loss of brush borders in the kidneys of Cre^+^/MCM4^fl/fl^ mice (Fig. 1E). A large number of cell fragments or necrotic epithelium were observed in the tubular lumen of Cre^+^/MCM4^fl/fl^ mice, accompanied by abnormal debris deposition in the tubular lumen, which resulted in lumen congestion (Fig. 1E). Moreover, the interstitial components and the infiltration of monocytes into the interstitium were also observed in the kidneys of Cre^+^/MCM4^fl/fl^ mice (Fig. 1E). All these pathological events were further aggravated in the right kidneys of Cre^+^/MCM4^fl/fl^ mice, where the right kidney was smaller than the left kidney. Toluidine blue staining revealed normal tubule morphology in the kidneys of Cre^−^/MCM4^fl/fl^ mice and disrupted the TBM in the kidneys of Cre^+^/MCM4^fl/fl^ mice, with columns of proliferating cells invading the interstitial space (Fig. S3C). These results suggest that MCM4 deficiency leads to severe tubular morphogenesis abnormalities, indicating a relatively uncommon but critical role of MCM4 in kidney development.

For a more in-depth analysis, renal tissues from Cre^−^/MCM4^fl/fl^ and Cre^+^/MCM4^fl/fl^ mice were used for transcriptome RNA-seq. We identified 5283 differentially expressed genes (DEGs) following MCM4 deficiency, including 3239 up-regulated and 2044 down-regulated genes (*P* < 0.05, fold change >1.5) (Fig. 1G). Genome pathway enrichment analysis showed that the up-regulated DEGs were associated with cell proliferation, inflammation response, and extracellular matrix (ECM) organization (Fig. S4A). Therefore, we verified these changes and found that the level of neutrophil gelatinase-associated lipocalin (NGAL), a known marker of tubular injury, was obviously increased in Cre^+^/MCM4^fl/fl^ mice, as further determined by immunoblot analysis (Fig. S4B). The up-regulation of F4/80 and Ki67 was also observed in Cre^+^/MCM4^fl/fl^ mice (Fig. 1H) with the increased ratio of apoptotic tubular cells (Fig. 1I). Notably, Cre^+^/MCM4^fl/fl^ mice showed a significantly more extensive Trichrome-Blue-positive matrix, consistent with more extensive interstitial fibrosis, as evidenced by the intense staining of vimentin and α-SMA in the interstitial area (Fig. 1J). Furthermore, immunoblot analysis further revealed a decrease in E-cadherin expression and an increase in the levels of fibronectin, vimentin, and α-SMA, suggesting that the loss of epithelial markers was associated with the increases in mesenchymal marker expression (Fig. S4C). These results further confirm the progressive evolution to a mesenchymal phenotype caused by MCM4 deficiency.

As shown in Figure S4D, pathway analysis showed that the enriched gene sets of the down-regulated DEGs were mainly involved in multiple metabolic processes, such as ATP metabolic process, tricarboxylic acid cycle, fatty acid metabolic process, lipid metabolic process, cholesterol homeostasis, steroid metabolic process, oxidation–reduction process, organic acid metabolic process, and carbohydrate metabolic process. MCM4 deletion appeared to disrupt various metabolic pathways, which may be associated with tubular injury, thereby affecting renal development.

More importantly, a decreased number of nephrons and collapsed tubular morphology were also observed as early as postnatal day 3 (P3) and P10. Compared to Cre^−^/MCM4^fl/fl^ mice, the kidneys of Cre^+^/MCM4^fl/fl^ mice decreased in size as early as P3 (Fig. S5A), and the difference was more noticeable at P10 (Fig. S5B). In addition to the above pathological changes in the renal tissue, renal cyst formation deserves more attention. As shown in Figure 1K, there were no significant tubular cysts present in 6-month-old Cre^−^/MCM4^fl/fl^ mice; however, dilated cystic tubules were evident in Cre^+^/MCM4^fl/fl^ mice of the same age. Moreover, cystic tubules were observed in the kidneys of the Cre^+^/MCM4^fl/fl^ mice as early as P10 and P40 (Fig. S5C). The contribution of renal cyst formation to MCM4 deficiency was verified via Ki67 staining. An increased number of Ki67-positive cells in the dilated cyst-lining epithelial cells was observed in the kidneys of Cre^+^/MCM4^fl/fl^ mice (Fig. S5D).

In summary, our study is the first to report that the loss of MCM4 in tubules leads to severe tubular morphogenesis abnormalities and renal failure, and even progresses to premature death in mice, consolidating the concept that MCM4 plays a critical role in renal dysplasia. Notably, MCM4 knockout mice exhibited severe renal dysplasia and early cystogenesis, which manifested as decreased nephron formation and disrupted tubular structure, followed by the subsequent evolution of the entire continuum of interstitial fibrosis and inflammation, which has important clinical implications. Taken together, our results indicate that MCM4 is a critical regulator of renal morphogenesis and cystogenesis, and may represent a novel pathogenic gene during renal development and provide insights into a therapeutic strategy for renal dysplasia. However, whether a therapeutic window exists for targeting MCM4 in patients with renal dysplasia remains to be determined in future studies.

## CRediT authorship contribution statement

**Jing Huang:** Writing – original draft, Methodology, Data curation, Software, Investigation, Conceptualization. **Hui-Ling Xiang:** Project administration, Investigation, Validation, Methodology, Data curation. **Qian Yuan:** Writing – review & editing, Supervision, Data curation, Validation, Project administration. **Chun Zhang:** Visualization, Funding acquisition, Writing – review & editing, Supervision, Conceptualization. **Xian-Fang Meng:** Writing – review & editing, Supervision, Conceptualization, Visualization, Project administration.

## Ethics declaration

All animals received care in compliance with the Principles of Laboratory Animal Care, and the study was approved by the Animal Ethics Committee of the Huazhong University of Science and Technology ([2022] IACUC Number: 2854). The procedures were conducted in accordance with the Guide for the Care and Use of Laboratory Animals of the National Institute of Health (Bethesda, MD, USA).

## Data availability

The authors declare that all data relevant to this study are available in the article or from the corresponding author upon reasonable request.

## Funding

This study was financially supported by the National Key Research and Development Program of China (No. 2024YFC3044900, 2021YFC2500200); the Key Research and Development Program of Hubei Province, China (No. 2023BCB034); and the National Natural Science Foundation of China (No. 82370728, 82500848, 82300786, 82300851).

## Conflict of interests

Chun Zhang is the member of Genes & Diseases Editorial Board. To minimize bias, he/she was excluded from all editorial decision-making related to the acceptance of this article for publication. The remaining authors declare no conflict of interests.
