# Supplementary material for: Loss of minichromosome maintenance 4 leads to early renal dysplasia and cystogenesis outcomes
Source: Genes Dis. 2025 Dec 8;13(5):101968. doi: 10.1016/j.gendis.2025.101968 (PMC13123473; doi:10.1016/j.gendis.2025.101968)
Supplement: Multimedia component 1 [file mmc1.docx]

**Supplemental Material**

**This file includes:**

**Supplementary Materials and methods**

**Supplementary Figures S1 to S5**

**Supplementary Materials and methods**

**Generation of tubule-specific MCM4 knockout mice**

The MCM4 conditional knockout mice (C57BL/6J), created via CRISPR/Cas9-mediated genome engineering (MCM4^fl/fl^ mice), were obtained from Cyagen Bioscience Inc. (Guangzhou, China). These mice were crossed with KSP-Cre mice (Cyagen Bioscience Inc.) to generate tubule-specific MCM4 knockout mice (Cre^+^/MCM4^fl/fl^ mice). Age-matched littermates homozygous for floxed MCM4 but lacking the Cre enzyme were used as controls (Cre^-^/MCM4^fl/fl^ mice). All animal experiments were approved by the Animal Ethics Committee of the Huazhong University of Science and Technology ([2022] IACUC Number: 2854). All procedures were performed in accordance with the Guide for the Care and Use of Laboratory Animals of the National Institutes of Health (Bethesda, MD, USA).

**Morphological analysis**

For morphological analysis, the kidney tissues were fixed in 4% paraformaldehyde, embedded in paraffin, and sectioned at a thickness of 4 µm. The sections were then stained with hematoxylin and eosin (HE) for morphological evaluation following the manufacturer's instructions (Beyotime, Shanghai, China). Fibrotic degree in the interstitial area was detected by Masson-trichrome staining following the manufacturer's instructions (Beyotime, Shanghai, China).

**Immunohistochemistry (IHC)**

Kidney tissue sections were incubated with primary antibodies overnight at 4 ℃ and then incubated with biotinylated secondary antibody for 1 h at 37 ℃. After staining with DAB, the density of the positively stained areas was calculated using Image-Pro Plus software. Sections were stained using the following antibodies: MCM4 (1:1000, ab4459, Abcam), neutrophil gelatinase-associated lipocalin (NGAL; 1:200, ab63929, Abcam), Ki67 (1:200, ab15580, Abcam), F4/80 (1:200, 70076, CST), vimentin (1:500, 5741, CST), and α-SMA (1:500, ab5694, Abcam).

**Immunofluorescence (IF) staining**

The frozen tissue sections were fixed with 4% paraformaldehyde for 30 min, permeabilized with 0.3% Triton X-100 for 20 min and blocked with 5% donkey serum for 1 h. Sections were immunostained with Lotus tetragonolobus lectin (LTL; 1:200, FL-1321, VECTOR Laboratories) or E-cadherin (1:200, 20874-1-AP, Proteintech) overnight at 4 °C, incubated with Alexa Fluor 488-conjugated donkey anti-rabbit (1:200, A32790, Invitrogen) or Alexa Fluor 594-conjugated donkey anti-mouse antibodies (1:200, A31573, Invitrogen) in the darkness for 1 h, and then counterstained with DAPI (Beyotime). Images were captured using an Olympus BX-53 microscope.

**Western blot**

Western blot was performed as previously described. Total protein from the kidney tissues was extracted using RIPA lysis buffer (Beyotime), separated via sodium dodecyl sulfate-polyacrylamide gel electrophoresis, and transferred to polyvinylidene fluoride membranes (Merck Millipore, MA, USA). Subsequently, the membranes were blocked with 5% non-fat milk for 1 h and incubated with primary antibodies overnight at 4 ℃. The densities of the images were analyzed using ImageJ software (National Institutes of Health, Bethesda, MD, USA). Primary antibodies against the following were molecules used: NGAL (1:1000, ab63929, Abcam), α-SMA (1:1000, ab5694, Abcam), Bax (1:2000, 50599-2-Ig, Proteintech), E-cadherin (1:2000, 20874-1-AP, Proteintech), fibronectin (1:5000, F3648, Sigma-Aldrich), vimentin (1:1000, 5741, CST), and GAPDH (1:5000, 10494-1-AP, Proteintech).

**RNA sequencing (RNA-seq) analysis**

Total RNA was extracted from the kidney cortex of MCM4^flox/flox^ and Ksp-cre/MCM4^flox/flox^ mice, and were subjected to RNA-seq analysis performed by MGI platform. The library construction, sequencing, and analysis were performed at Wuhan SEQHEALTH Co. Ltd (Wuhan, China).

**Terminal deoxynucleotidyl transferase-mediated dUTP nick and labeling (TUNEL) assay**

Apoptotic cells in frozen tissue sections were measured using an *in situ* Apoptosis Detection kit (Roche, Mannheim, Germany), as described previously [28]. The sections were fixed with 4% paraformaldehyde for 20 min, permeabilized with 0.3% Triton X-100 for 20 min, and incubated with the TUNEL reaction mixture for 1 h at 37 ℃ in darkness. Images were acquired using a fluorescence microscope (Olympus).

**Statistical analyses**

All data in this study are represented as means ± SEM and were analyzed and graphed using GraphPad Prism 8.0 software (La Jolla, USA). A two-tailed, unpaired Student’s t-test was used to compare the differences between two groups, and *P* < 0.05 was considered statistically significant.

**Supplementary Figures**

**Figure S1**

**
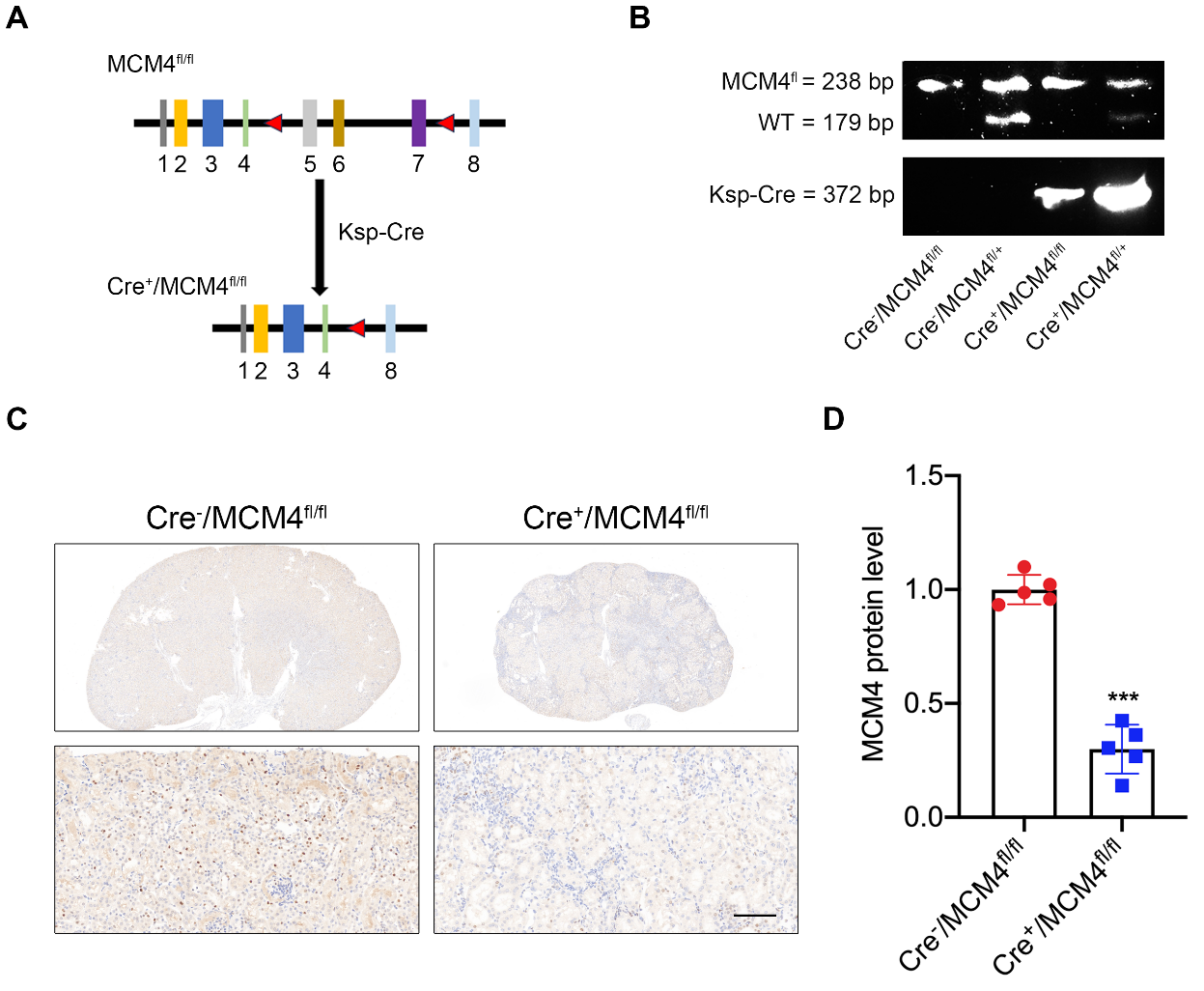
**

**Figure S1.** **Generation of** **tubule-specific MCM4 knockout mice.** **(A)** Generation of conditional knockout mice with specific ablation of MCM4 in proximal tubular epithelial cells using the Cre-LoxP recombination system. **(B)** Genotyping confirmation of conditional knockout through tail preparation and polymerase chain reaction (PCR) at 2 weeks of age. **(C-D)** Representative immunohistochemistry (IHC) images and quantification of MCM4 expression in two groups. Upper scale bar: 500 μm, lower scale bar: 50 μm. N = 5 for each group. All data were represented as mean ± SEM. ****P* < 0.001 *vs.* Cre^-^/MCM4^fl/fl^.

**Figure S2**


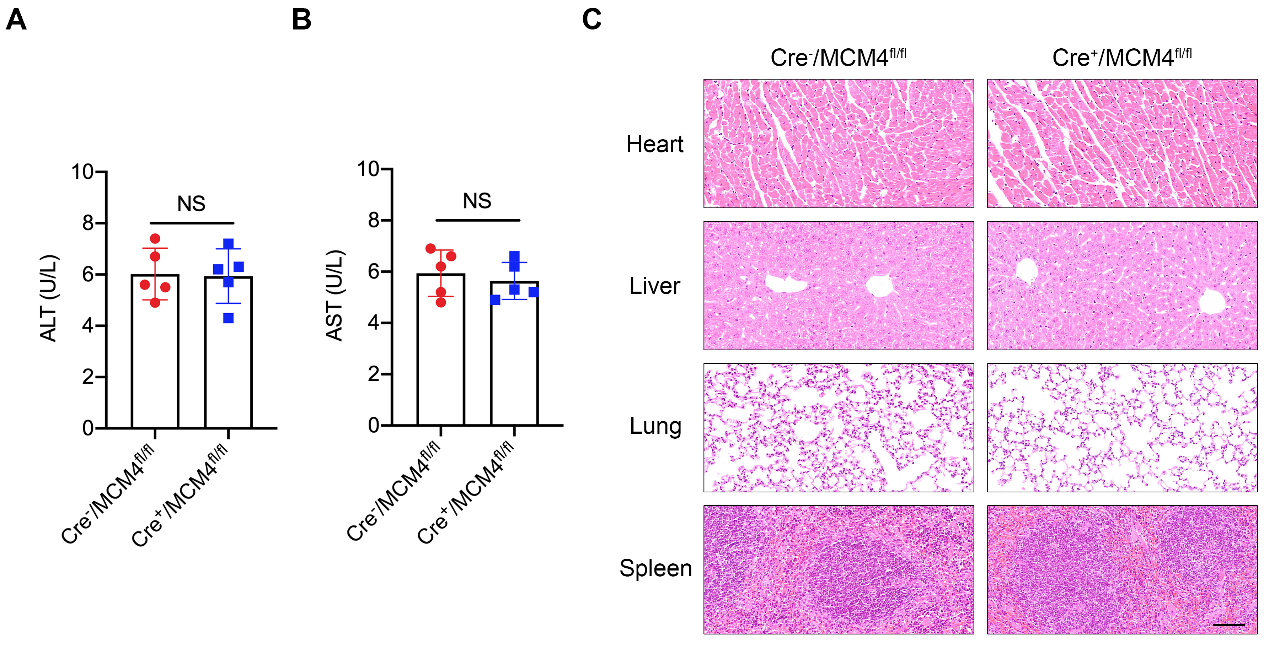


**Figure S2. Loss of MCM4 in tubule had no effect on other organs. (A)** Graphical representation of the levels of alanine aminotransferase in two groups at 40 days. **(B)** Graphical representation of the levels of aspartate aminotransferase in two groups at 40 days. **(C)** Hematoxylin and eosin staining of heart, liver, lung, and spleen of mice in two groups at 40 days. Scale bar: 50 μm. N = 5 for each group. All data were represented as mean ± SEM.

**Figure S3**


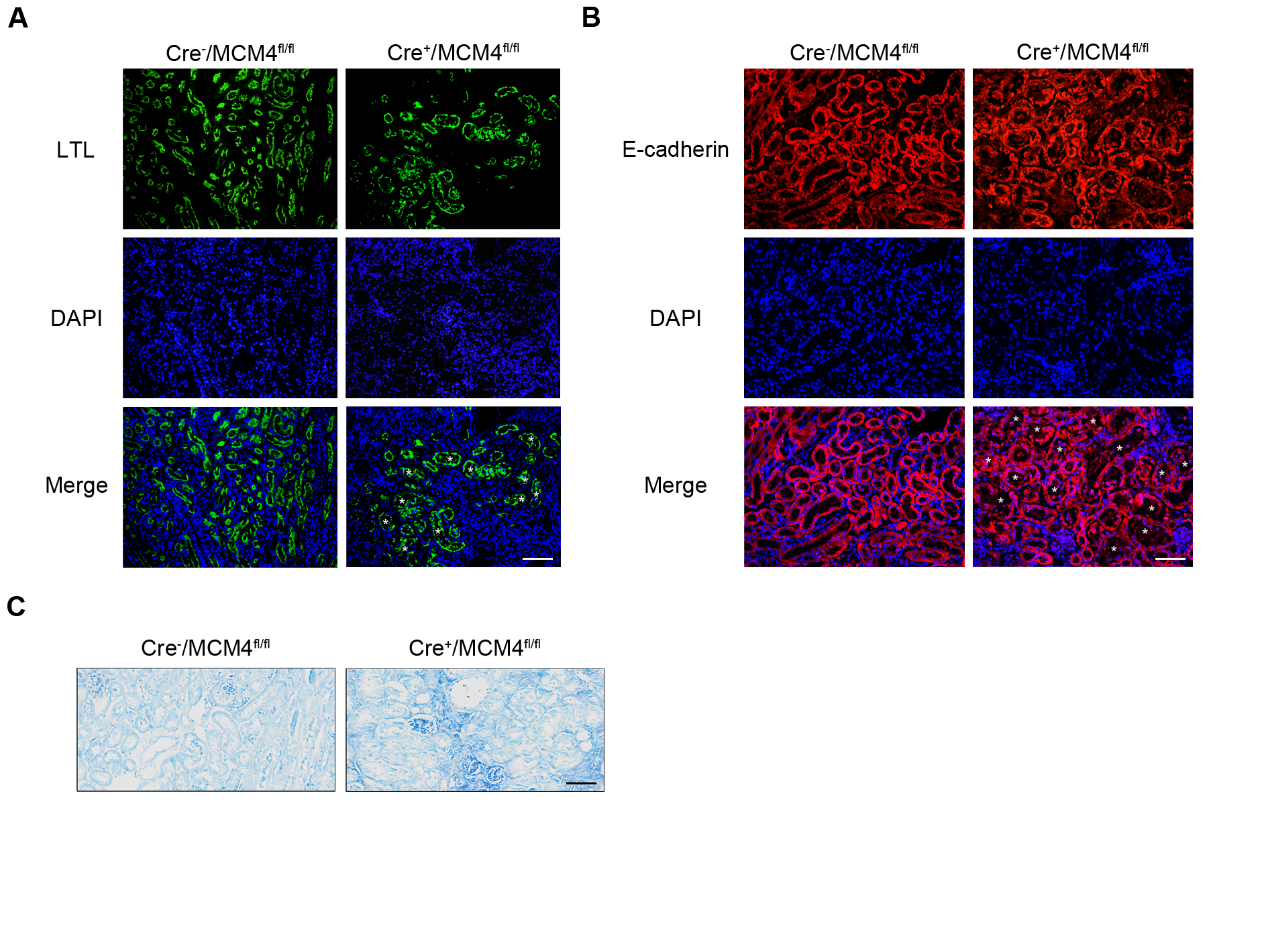


**Figure 3.** **Loss of MCM4 in tubule reduced the number of tubules and destroyed tubular structure in the kidney. (A)** Immunofluorescence staining of LTL in kidney of mice in two groups at 40 days. Scale bar: 50 μm. **(B)** Immunofluorescence staining of E-cadherin in kidney of mice in two groups at 40 days. Scale bar: 50 μm. **(C)** Semithin toluidine blue-stained sections showing the disrupted TBM in Cre^+^/MCM4^fl/fl^ mice. Scale bar: 50 μm. N = 5 for each group.

**Figure S4**


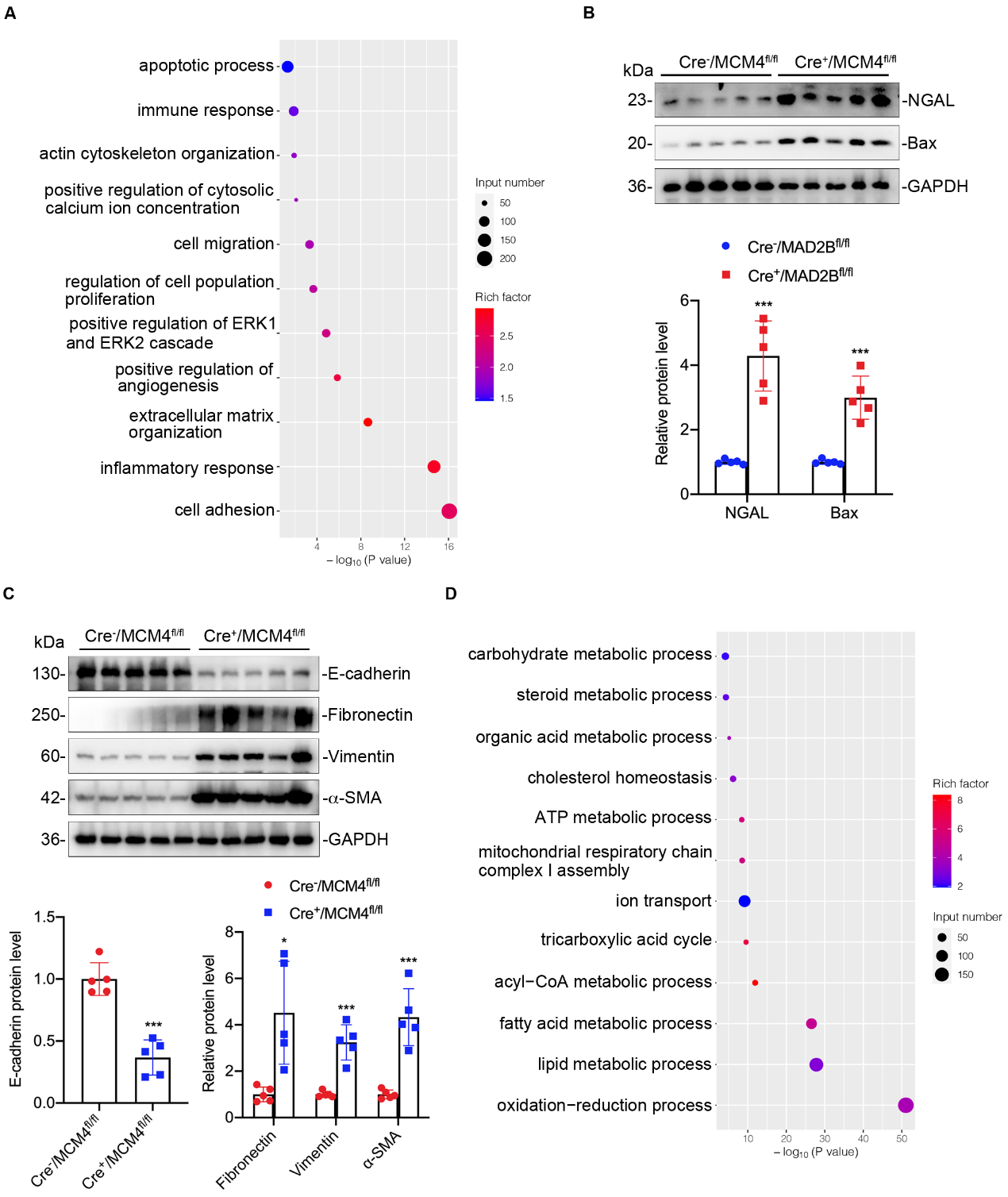


**(A)** Enriched gene sets of upregulated DEGs in MCM4 knockout mice by RNA-seq analysis. **(B)** Representative western blots and summarized data showing the renal expression of NGAL and Bax in two groups of mice at 40 days, as indicated. **(C)** Representative western blots and summarized data showing the renal expression of E-cadherin, fibronectin, a-SMA, and vimentin in two groups of mice at 40 days, as indicated. **(D)** Enriched gene sets of downregulated DEGs in MCM4 knockout mice by RNA-seq analysis. N = 5 for each group. All data were represented as mean ± SEM. **P* < 0.01, ****P* < 0.001 *vs.* Cre^-^/MCM4^fl/fl^.

**Figure S5**


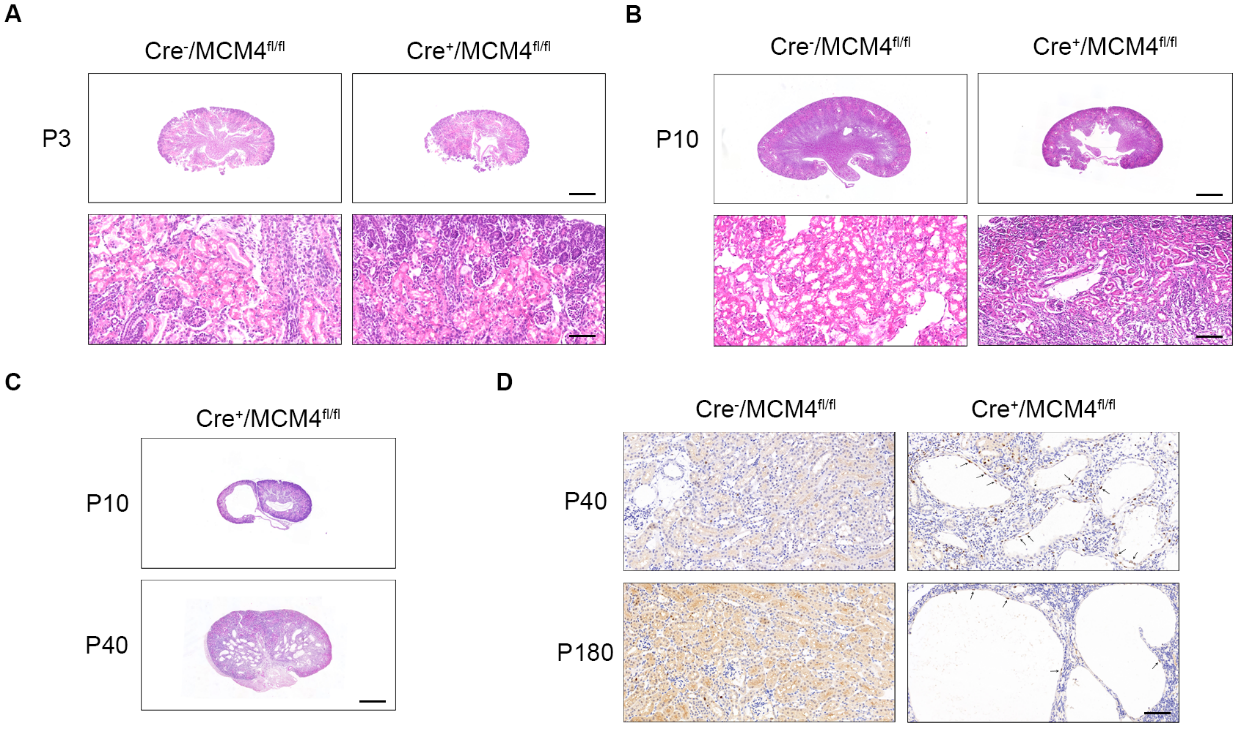


**Figure S5. Contribution of MCM4 in early stage of renal dysplasia and cystogenesis. (A)** HE staining of kidneys of mice in two groups at postnatal 3 days. Upper scale bar: 500 μm, lower scale bar: 50 μm. **(B)** HE staining of kidneys of mice in two groups at postnatal 10 days. Upper scale bar: 500 μm, lower scale bar: 50 μm. **(C)** HE staining showing cystic kidneys in Cre^+^/MCM4^fl/fl^ mice at postnatal 10 days and 40 days. Scale bar: 500 μm. **(D)** Representative immunostaining of Ki67 in kidney of mice in two groups at postnatal 40 days and 6 months. Scale bar: 50 μm. N = 5 for each group.
